# Microbiological, Histological, Immunological, and Toxin Response to Antibiotic Treatment in the Mouse Model of *Mycobacterium ulcerans* Disease

**DOI:** 10.1371/journal.pntd.0002101

**Published:** 2013-03-14

**Authors:** Fred Stephen Sarfo, Paul J. Converse, Deepak V. Almeida, Jihui Zhang, Clive Robinson, Mark Wansbrough-Jones, Jacques H. Grosset

**Affiliations:** 1 Komfo Anokye Teaching Hospital, Kumasi, Ghana; 2 Johns Hopkins University Center for Tuberculosis Research, Baltimore, Maryland, United States of America; 3 St. George's, University of London, London, United Kingdom; University of Tennessee, United States of America

## Abstract

*Mycobacterium ulcerans* infection causes a neglected tropical disease known as Buruli ulcer that is now found in poor rural areas of West Africa in numbers that sometimes exceed those reported for another significant mycobacterial disease, leprosy, caused by *M. leprae*. Unique among mycobacterial diseases, *M. ulcerans* produces a plasmid-encoded toxin called mycolactone (ML), which is the principal virulence factor and destroys fat cells in subcutaneous tissue. Disease is typically first manifested by the appearance of a nodule that eventually ulcerates and the lesions may continue to spread over limbs or occasionally the trunk. The current standard treatment is 8 weeks of daily rifampin and injections of streptomycin (RS). The treatment kills bacilli and wounds gradually heal. Whether RS treatment actually stops mycolactone production before killing bacilli has been suggested by histopathological analyses of patient lesions. Using a mouse footpad model of *M. ulcerans* infection where the time of infection and development of lesions can be followed in a controlled manner before and after antibiotic treatment, we have evaluated the progress of infection by assessing bacterial numbers, mycolactone production, the immune response, and lesion histopathology at regular intervals after infection and after antibiotic therapy. We found that RS treatment rapidly reduced gross lesions, bacterial numbers, and ML production as assessed by cytotoxicity assays and mass spectrometric analysis. Histopathological analysis revealed that RS treatment maintained the association of the bacilli with (or within) host cells where they were destroyed whereas lack of treatment resulted in extracellular infection, destruction of host cells, and ultimately lesion ulceration. We propose that RS treatment promotes healing in the host by blocking mycolactone production, which favors the survival of host cells, and by killing *M. ulcerans* bacilli.

## Author Summary

*Mycobacterium ulcerans* infection causes Buruli ulcer (BU), a disfiguring skin disease now found principally in poor rural areas of West Africa. *M. ulcerans* produces a toxin called mycolactone (ML), which destroys fat cells in skin tissue. BU typically first shows as a nodule that eventually ulcerates. The lesions may continue to spread over limbs or occasionally the trunk. The current standard treatment is 8 weeks of daily rifampin and injections of streptomycin (RS). The treatment kills the bacilli and wounds gradually heal. We tried to determine if RS treatment actually stops mycolactone production before killing bacilli. Using a mouse footpad model of *M. ulcerans* infection where the time of infection and lesion development can be followed in a controlled manner before and after antibiotic treatment, we found that RS treatment rapidly reduced footpad swelling, *M. ulcerans* numbers, and ML production. Microscopic analysis of footpads revealed that RS treatment resulted in bacilli being destroyed by host cells whereas lack of treatment resulted in extracellular infection, destruction of host cells, and lesion ulceration. We propose that RS treatment promotes healing in the host by blocking mycolactone production, which favors the survival of host cells, and by killing *M. ulcerans*.

## Introduction

*Mycobacterium ulcerans* infection is the cause of the neglected tropical disease, Buruli ulcer, found in poor rural areas of West Africa as well as in beach resorts in Australia [1] principally, although transmission has occurred in every continent except Europe. It is the third most important mycobacterial infection of humans worldwide after tuberculosis and leprosy, although in Benin and Ghana, for example, it is now the second most common mycobacterial disease [2], [3]. Infection begins after exposure in slow-moving fresh water to plants or biting insects or other unknown mechanisms and slowly leads to swelling, manifested as a nodule, plaque, or edema in humans, and in experimental animals [4], [5], [6], [7], [8]. Untreated lesions may progress and involve an entire limb or the trunk. Part of the pathogenic process is the production of the immunosuppressive mycolactone (ML) toxin by *M. ulcerans* that promotes the development of necrotic ulcers and possibly the initial swelling. How soon ML production begins after infection is unknown [9] as is how soon it stops due to antibiotic treatment, currently rifampin and streptomycin [1], although studies of human lesions have suggested production may be affected soon after treatment [10].

Evidence from the mouse model indicates that BALB/c mice develop swelling accompanied by increasing bacterial burden as measured by CFU followed by a plateau in CFU but progression of swelling [4], [11], [12]. After antibiotic treatment, both swelling and CFU decline [13].

We evaluated the evolution of *M. ulcerans* (Mu1615, Malaysian strain) infection in the mouse footpad model by assessing not only lesion appearance and CFU number, but also ML production, systemic immune responses, and histopathology at different times after infection and after the onset of antibiotic therapy. We find that the current standard antibiotic therapy started after the appearance of swelling not only reduces bacterial load but also preserves an effective host cell infiltrate leading to loss of acid-fast stain integrity of the bacilli. The host immune response was tested in splenocytes stimulated with mycobacterial antigens and assessed by measuring cytokine and chemokine production. Additionally, there is an early and continuing restraint on ML production that may help the host control and reduce bacillary numbers.

## Materials and Methods

### Bacteria

*M. ulcerans* 1615 (Mu1615), an isolate originally obtained from a patient in Malaysia in the 1960s, [14] was kindly provided by Dr. Pamela Small, University of Tennessee. Previous studies have confirmed that this strain produces mycolactone and kills macrophages and fibroblasts [15], [16]. The strain was passaged in mouse footpads before use in these studies. The bacilli were harvested from swollen footpads at the grade 2 level, i.e., swelling with inflammation of the footpad [4].

### Infection and CFU/histopathology/immunology analyses

BALB/c mice, age 4–6 weeks (Charles River, Wilmington, MA), were inoculated in the right hind footpad with approximately 5.5 log_10_ (3×10^5^) CFUs of Mu1615 in 0.03 ml PBS. Footpads were harvested from 9 mice (3 for CFU count, 3 for ML detection, 3 for histopathology) at different time points after infection (Table 1) up to ≥grade 3 swelling. After the onset of swelling (∼day 21–23), treatment with rifampin (R, Sigma, St. Louis, MO, 10 mg/kg by gavage) and streptomycin (S, Sigma, 150 mg/kg by subcutaneous injection) began on day 24, and was administered 5 days/week for 8 weeks. Groups of the treated mice were also sacrificed for these analyses. For CFU counts footpad tissue was harvested, minced with fine scissors [17], suspended in 2.0 ml PBS, serially diluted, and plated on Middlebrook selective 7H11 plates (Becton- Dickinson, Sparks, MD). Alternatively, footpads were harvested and placed in 10% buffered formalin for histopathological analysis by hematoxylin and eosin (H&E) or acid-fast (AF) staining. Footpads from a third group of mice at each time point were placed in freezer vials and frozen at −80°C. These latter footpads were shipped on dry ice to St. George's University of London for mycolactone detection experiments. Mice were evaluated for footpad swelling weekly using an established scoring system [4] with grade 1 showing footpad swelling, grade 2 swelling with inflammation, and grade 3 swelling and inflammation of the entire foot [18]. Spleens were harvested to assess responses to mycobacterial culture filtrate proteins (CFP) and to the concanavalin A (ConA) mitogen (Sigma) by multiplex ELISA (Bio-Plex Pro Mouse Cytokine 23-plex Assay, Biorad, Hercules, CA) as described [15]. The 23 analytes included Th1 cytokines: IL-2, IFN-γ, IL-12p40, IL-12p70; Th2 cytokines: IL-4, IL-5, IL-10, and IL-13; proinflammatory cytokines: TNF-α, IL-1α, IL-1β, and IL-6; IL-9; IL-17; colony stimulating factors: GMCSF, GCSF, IL-3; and chemokines: CXCL-1, CCL-2, CCL-3, CCL-4, CCL-5, and CCL-11. All animal procedures were conducted according to relevant national and international guidelines. The study was conducted adhering to the Johns Hopkins University guidelines for animal husbandry and was approved by the Johns Hopkins Animal Care and Use Committee, protocol MO08M240. The Johns Hopkins program is in compliance with the Animal Welfare Act regulations and Public Health Service (PHS) Policy and also maintains accreditation of its program by the private Association for the Assessment and Accreditation of Laboratory Animal Care (AAALAC) International.

**Table 1:** Scheme of study.

Day	3	7	13	20	27	34	40	48	55	62	70	78	98	Total
**Control mice**														
CFU	3	3	3	3	3	3	3	3	3					27
Mycolactone	3	3	3	3	3	3	3	3	3	3				30
Histopathology	3	3	3	3	3	3	3	3	3					27
**Treated mice**														
CFU					3	3	3	3	3	3		3	3	24
Mycolactone					3	3	3	3	3	3				18
Histopathology					3	3	3	3	3				3	18
**Total**	9	9	9	9	18	18	18	18	18	9		3	6	144

Control and treated (i.e., Rifampin, 10 mg/kg, and streptomycin, 150 mg/kg) mice were sacrificed at the indicated days after infection for CFU analysis, mycolactone detection, immunological assessment, or histopathology. Treatment began on day 24 after infection.

### Mycolactone detection assays

#### Extraction of lipids from mouse footpads for mycolactone detection and quantification

Mouse footpads were weighed, diced with a scalpel blade and homogenised in 500 µl extraction solution of chloroform: methanol 2∶1 vol/vol with ceramic beads in a Fastprep ribolyser at power 6.5 for 45 seconds and again at power 5.0 for 45 seconds [19]. After homogenisation, an additional 500 µl of extraction solution was added and samples were placed on ice for 4 hours. 0.2 ml of 0.3% sodium chloride solution was added for Folch's extraction and samples were centrifuged at 13,000 g for 5 minutes. The organic phase was harvested, dried down under vacuum and lipids re-suspended in ice-cold acetone for an hour and centrifuged at 13,000 g for 5 minutes to remove phospholipids. Acetone soluble lipids (ASL) were dried down under vacuum and re-suspended in 100% ethanol.

#### Cytotoxicity assay for mycolactone quantification

Cytotoxicity of ASL from infected mouse tissue was tested using the MTT assay, a colorimetric test assessing cellular viability, as previously described [20], [21]. Briefly, HELF cells (kindly donated by Dr. Kay Capaldi) were maintained in Dulbecco's modified Eagle's medium supplemented with 10% fetal calf serum and 2 mM L-glutamine in the presence of penicillin and streptomycin 100 mU/ml and 100 mg/ml respectively and incubated in 5% carbon dioxide at 37°C. For cytotoxicity assays proliferating HELF cells were seeded at a density of 10^5^/well in microtiter plates overnight.

ASL were dissolved in 100 µl of absolute ethanol and 2-fold dilutions performed up to 1∶32. 5 µl of each dilution was used to treat HELF cells in triplicate. After incubation for 48 hrs, 20 µl of 5 mg/ml of MTT (Sigma) was added to each well and incubated for a further 4 h for purple colored formazan crystals to develop following which 100 µl of detergent solution of isopropanol∶HCl (2N) in a ratio of 49∶1 was used to dissolve formazan crystals for spectrophotometric quantification in a multiplate well reader at 570–690 nm. Time course and dose-response curves were derived by treating HELF cells with serial dilutions of purified mycolactone A/B from 250 ng/ml to 0.3 ng/ml (kind gift of Professor Y. Kishi, Harvard University, USA]. All calibration and quantification experiments were performed at least twice. Concentration of mycolactone was determined within the linear portion of the dose response curve since cytotoxicity followed a sigmoidal dose-response relationship.

#### Mass spectroscopy

Serial dilutions of synthetic mycolactone A/B standards from 80 ng/ml to 1.25 ng/ml and ethanolic lipid extracts diluted 1∶10 in 96-well plates (Nunc) and liquid chromatographic separation and tandem mass spectrometric detection were performed using an ABI Sciex 3200 Q Trap mass spectrometer (ABI Sciex, UK) interfaced with a Shimadzu UFLC system (UFLC XR system with CBM-20A controller, SIL-20AC XR Prominence autosampler, CTO-20AC Prominence column oven, LC-20AD XR and LC-20 AD pumps (Shimadzu, UK)).

Mycolactone A/B was characterized by Enhanced Product Ion (EPI) detection. EPI conditions were optimized by infusing a 1 µg/mL ethanolic solution of mycolactone A/B standard via a Harvard syringe pump at flow rate of 10 µL/min. Characteristic ions in mycolactone A/B EPI spectra were m/z 765.5 [M+Na^+^], 429.3 and 359.2. EPI parameters for mycolactone A/B were: declustering potential (DP) 20V, entrance potential (EP) 10V, collision cell entrance potential (CEP) 32.35V and collision energy (CE) 65 eV.

Mycolactone was quantified by Multiple Reaction Monitoring (MRM). MRM transitions of mycolactone were identified by the compound optimization tool in Analyst 1.5 during the infusion of 1 µg/mL mycolactone A/B standard. From these, MS-MS fragmentation of the mycolactone A/B sodium adduct [M+Na^+^] (m/z 765.5, Q1 mass) to m/z 429.3 (Q3 mass) was selected for quantification. MRM parameters were dwell time 150 ms, DP 126V, EP 10V, CEP 34V, CE 57 eV, cell exit potential (CXP) 6V. Source parameters for LC-MS-MS were: curtain gas 30 psi, collision gas medium, ion spray voltage 5500 V, temperature 400°C, nebulizer gas 30 psi, turbo gas 40 psi.

For the analysis of lipid extracts from mouse footpads, UPLC was combined with EPI methods (LC-EPI) or MRM methods (LC-MRM). Solvent A was 0.1% formic acid and 10-µM ammonium acetate in water. Solvent B was 0.1% formic acid and 10-µM ammonium acetate in acetonitrile. Samples (10 µL) were injected via autosampler and eluted at 0.5 mL/min on a Shima-Pack XR-ODS II column (2.1 µm, 2.0 mm×75 mm) by a 20–98% linear gradient of B over 2 min. Solvent composition was then held for 4 min before returning to the starting conditions. Standard curves (1.25 ng/mL to 100 ng/mL) were prepared using ethanolic stock solutions of synthetic mycolactone A/B.

### Statistical analysis

GraphPad Prism 4 was used to assess significant differences by student T and analysis of variance.

## Results

### Footpad swelling and CFU counts before and after treatment

**Figure 1:**
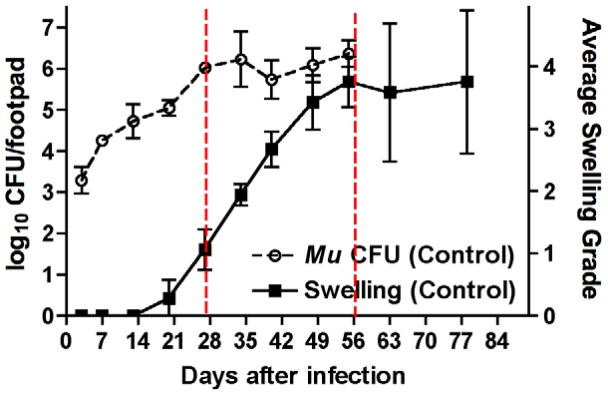
After reaching plateau in bacillary numbers, *M. ulcerans*-infected mouse footpads continue to increase in swelling. Swelling first became apparent at day 20 after infection. For the first 3 weeks, the number of cultivable organisms increased from 3.29±0.33 log_10_ on day 3 to 4.26±0.08 log_10_ on day 7, 4.73±0.42 log_10_ on day 13, 5.05±0.19 log_10_ on day 20, and 6.02±0.13 log_10_ on day 24, the first day of treatment. CFU remained at approximately this level for the remainder of the experiment in untreated mice. Swelling averaged ∼grade 1 at day 27 after infection, nearly grade 2 at day 34, grade 2.67 at day 40, 3.42 at day 48, 3.75 at day 55, 3.58 at day 63, and 3.75 again at day 78. Data based on 3 mice per group per time point. Red lines emphasize the period between the onset and augmentation of swelling and the plateau in CFU.

**Figure 2:**
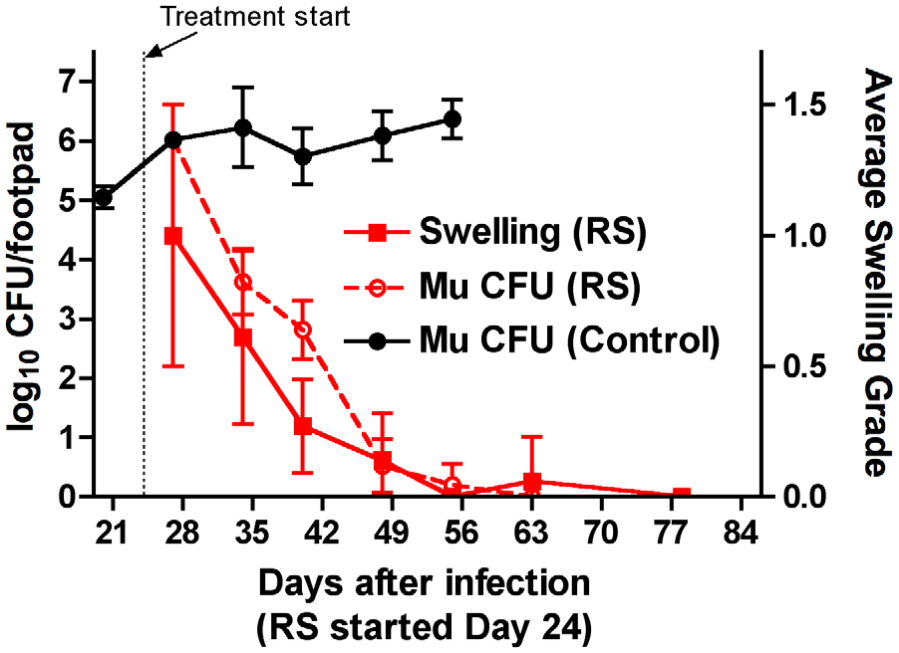
Swelling and CFU Reduction after RIF-STR treatment. After 3 days of RIF-STR (RS) treatment (day 27 after infection), swelling averaged grade 1, and then declined to 0.61±0.33, 0.27±0.18, 0.14±0.18, 0, 0.06±0.17, and 0 on days 34, 40, 48, 44, 63, and 78, respectively. CFU also declined from 6.02±0.09 log_10_ on day 27, and then 3.83±0.56 log_10_, 2.82±0.49 log_10_, 0.52±0.45 log_10_, 0.2±0.35 log_10_, 0, and 0 on days 34, 40, 48, 44, 63, and 78, respectively. For comparison, CFU per footpad for untreated controls (black line) show the impact of antibiotic treatment on bacterial burden. Data based on 3 mice per group per time point.

Three days after infection, mice harbored 3.29±0.33 log_10_ CFU in the infected footpads. Just before the onset of swelling, at day 20, the footpads contained 5.05±0.19 log_10_ CFU of *M. ulcerans*. Footpad swelling first appeared in some mice 3 weeks after infection and averaged grade 1 soon after the initiation of treatment at ∼3.5 weeks after infection. At day 27, there were approximately 6.02±0.13 log_10_ CFU per footpad. Swelling continued to increase over the following 3 weeks (Figure 1) for an average of grade 3.42±0.43 in untreated mice whereas CFU counts remained essentially unchanged. In contrast, both swelling and CFU counts declined markedly in the RS-treated mice. Swelling after 3 weeks of treatment averaged grade 0.14±0.18 and log_10_ CFU count per footpad was reduced to 0.52±0.45. By day 55 after infection, untreated mice had 6.37±0.32 log_10_ CFU whereas RS-treated mice, at this time point after 31 days of treatment, had only 0.20±0.35 log_10_ CFU (Figure 2a). By day 63 (i.e., 39 days of treatment), the RS-treated mice were culture negative. Swelling was essentially undetectable. Figure 2b shows the contrast in *M. ulcerans* CFU counts between treated and untreated mice.

### Histopathological assessment of *M. ulcerans* in lesions before and after treatment

**Figure 3:**
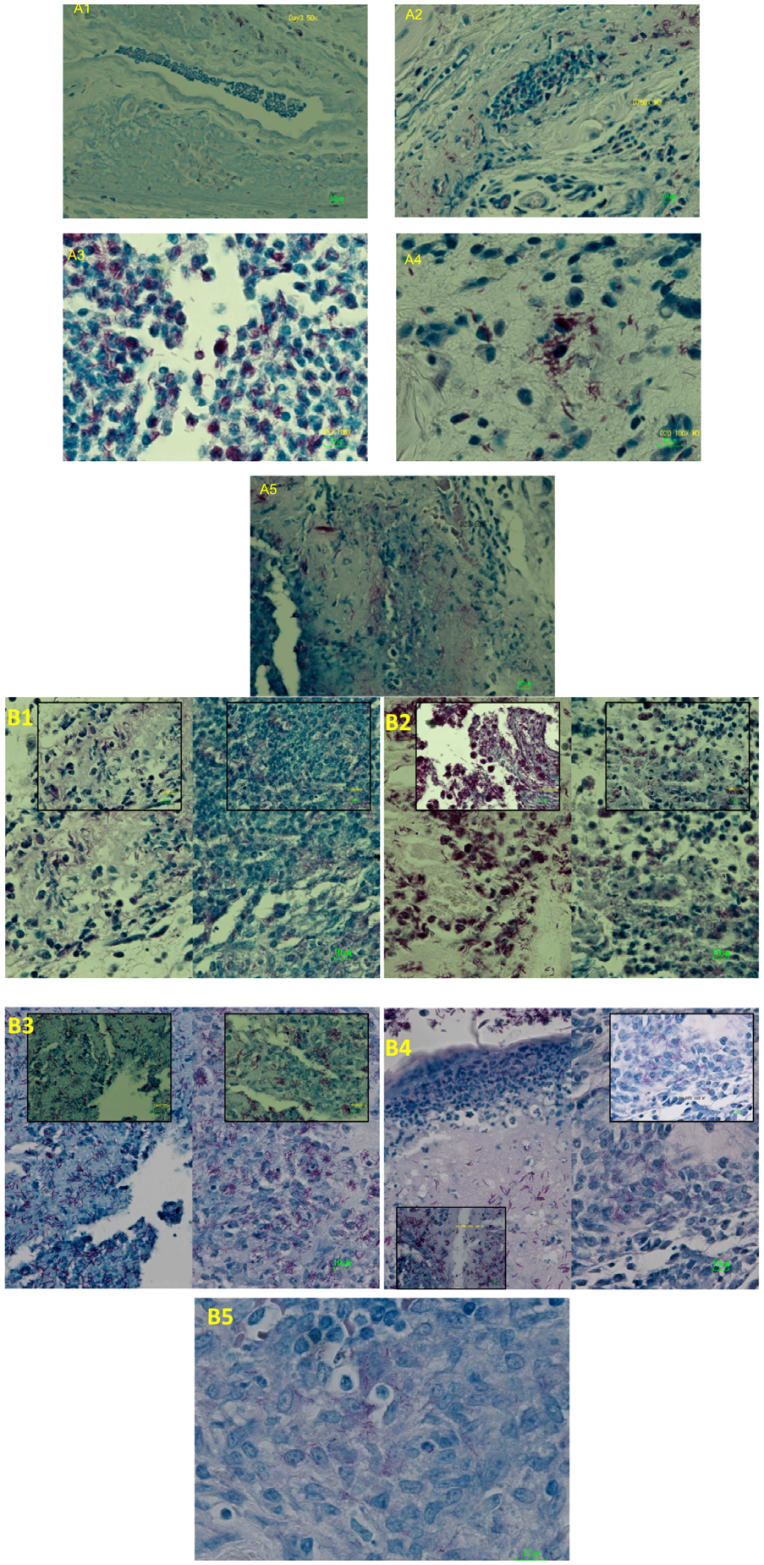
*M. ulcerans* in mouse footpads, before (A, 1–5) and after (B, 1–5) treatment with Rifampin-Streptomycin. A1: AFB are detectable in the dermis 3 days after infection with little or no cellular infiltrate. 500× Magnification A2: At day 7, the migration of inflammatory cells and phagocytosis has begun. 500× Magnification A3: By day 13, AFB are primarily associated with inflammatory and phagocytic cells. 1000× Magnification A4: At day 20, the extracellular phase of *M. ulcerans* infection has begun, coinciding with the onset of footpad swelling. 1000× Magnification A5: At day 27, shortly after the initiation of antibiotic treatment, a mix of intracellular and extracellular AFB is observed. 500× Magnification In panel B, untreated footpad lesions are shown on the left and treated footpads are shown on the right. B1: At day 34 after infection and after 10 days of treatment, *M. ulcerans* bacilli in treated mice are primarily associated with host cells whereas the bacilli are outside of cells in untreated mice; the cells are either not recruited or may have been destroyed by the mycolactone toxin. 500×, inset 1000×, Magnification, B2: At day 40, bacilli are found in extracellular masses in untreated mice but in mice treated for 16 days, the bacilli are still associated with host cells and stain less solidly. 500×, inset 1000×, Magnification B3: At day 48, after 24 days of treatment, the bacilli have lost solid staining and have become beaded in appearance. 500×, inset 1000×, Magnification B4: At day 55 after infection, *M. ulcerans* bacilli are apparent in the superficial dermis and epidermis and are being shed from the ulcerated lesions of untreated mice. 500×, inset 1000×, Magnification B5: 3 months after treatment completion, the organisms are uncultivable but non-solid staining bacilli are still present in the footpads. 500× Magnification.

Histologically, increased numbers of AFB correlated with increased footpad swelling in untreated mice; bacilli were evident initially in the dermis and eventually in sub-epidermal zones and the epidermis with the onset of ulceration (Figure 3A.1–5). Inflammatory cell infiltrates appeared to be maintained in RS-treated mice but were disrupted and progressively disorganized in untreated, control mice (Figure 3B.1–4). After 2–3 weeks of treatment, AFB were still detectable but acquired a beaded appearance and this morphology persisted even after treatment completion. The beaded appearance of the bacilli correlated with reduced numbers of cultivable bacilli in treated mice (Figure 2) whereas the bacilli in untreated mice appeared solidly stained. Three months after treatment completion, bacilli were detectable in tissue by acid-fast staining but were not cultivable (Figure 3B.5).

### Evolution of immune responses during the course of *M. ulcerans* infection

**Figure 4:**
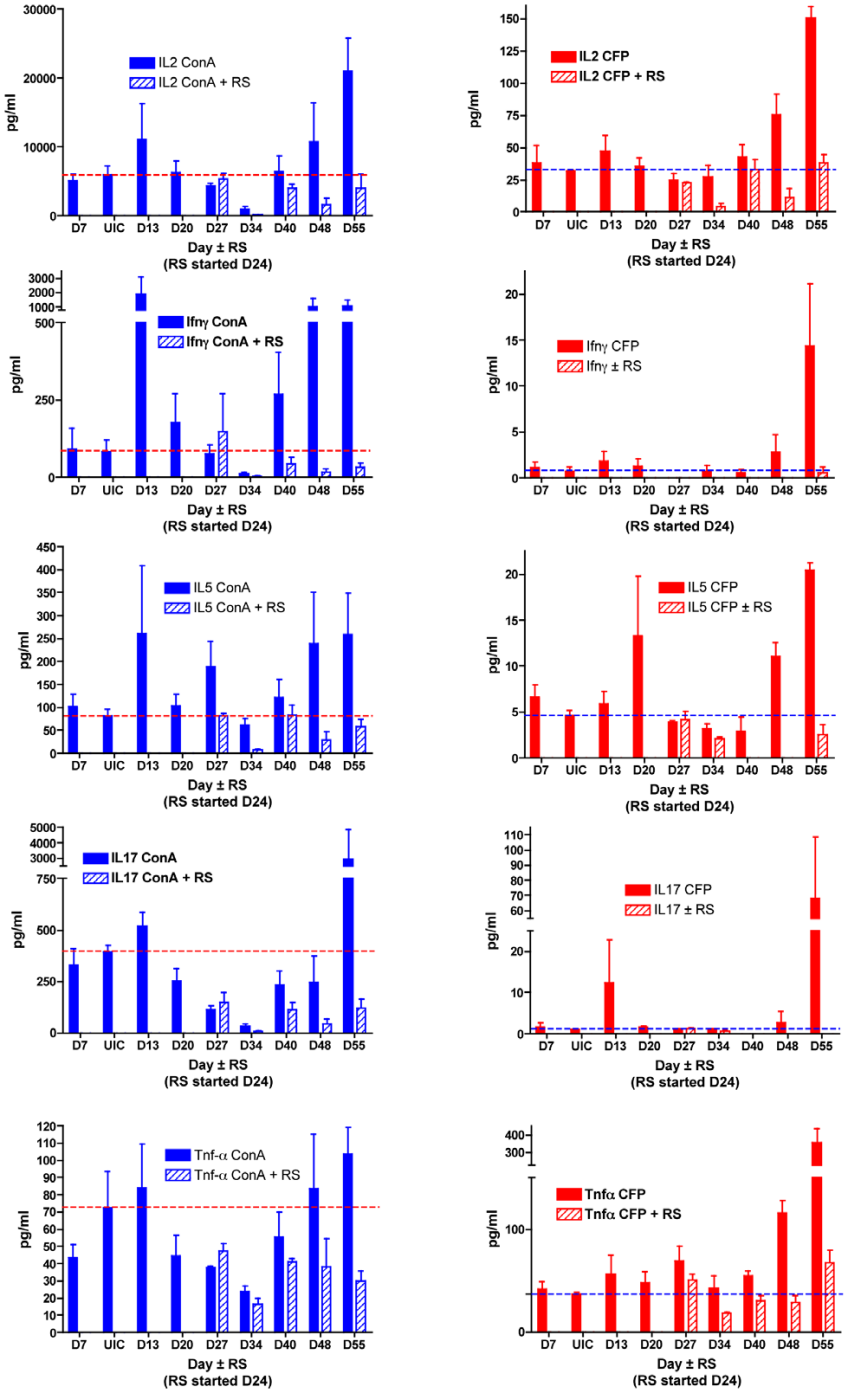
Cytokine production responses to concanavalin A or mycobacterial protein before and after Rifampin-Streptomycin treatment. The dotted line indicates the levels in uninfected control (UIC) mice. Responses by untreated mice are indicated by solid bars and those by rifampin-streptomycin (RS) treated mice are indicated by hatched bars. Responses of splenocytes stimulated with Concanavalin A (ConA) are in the left column and with mycobacterial culture filtrate proteins (CFP) in the right column for representative cytokines, from top to bottom, IL-2, IFN-γ, IL-5, IL-17, and TNF-α. Data based on 3 mice per group per time point.

ConA and CFP of *M. tuberculosis* (Mtb) were used as a potent T-cell mitogen and a non-specific mycobacterial antigen, respectively, to both assess the functional capability of T-cells and the evolution of cellular immune responses by splenocytes to mycobacterial antigens during the course of *M. ulcerans* infection. There is a biphasic pattern to the kinetics of secretion of representative Th-1 (IFN-γ), Th-2 (IL-5) and Th-17 (IL-17) cytokines upon stimulation with the potent T-cell mitogen ConA (Figure 4, left column). There is an initial rise detected at week 2, then a decline during weeks 4 to 6 and then a later surge from week 7 onwards. The cellular responses to a culture filtrate protein of Mtb however is generally a slowly evolving one and gradually builds up (IL-2, IL-5, IFN-γ and TNF-α) over the course of infection with *M. ulcerans* (Figure 4, right column). Immune responses were, however, generally lower during the course of infection in mice treated with antibiotics compared with untreated mice (Figure 4). Although the amount of TNF-α detected in supernatants of splenocytes from mice treated with RS was much less after 10 days of treatment, it was statistically significant (p<0.001) only at day 55 by two-way ANOVA. A t-test analysis indicated borderline statistical significance (p<0.05) at day 48.

### The presence of mycolactone in footpads before and after treatment

Mycolactone toxin production was assessed by two different methods: cytotoxicity against the HELF cell line and mass spectrometric analysis.

#### Cytotoxicity assay of lipid extracts from mouse footpads

**Figure 5:**
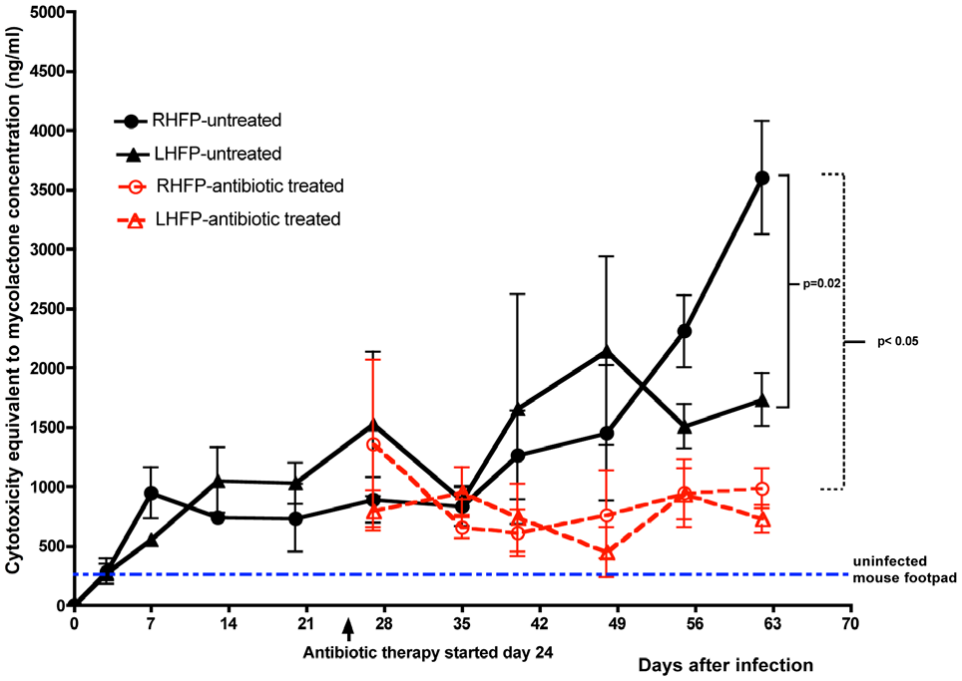
Cytotoxic activity of lipids extracted from footpads of mice without treatment or treated with rifampin-streptomycin. Cytotoxicity of lipid extracts was measure by the MTT assay after incubation with HELF cells for 48 hrs. Cytotoxicity is expressed relative to that induced by synthetic mycolactone measured concurrently. Data based on 3 mice per group per time point. By day 63 there were statistically significant differences between the infected right hind footpad (RHFP, black solid circles) and the contralateral (LHFP, solid diamonds, p<0.02) and footpads of the rifampin-streptomycin (Abx) treated mice (RHFP, red open circles; LHFP, red open diamonds p<0.05).

Lipid extracts from the right footpads into which *M. ulcerans* had been injected were increasingly cytotoxic from day 3 up to day 62. Surprisingly, lipid extracts from the left footpad of the same mice also demonstrated significant cytotoxicity (Figure 5). Lipid extracts from footpads of mice which had not been infected with *M. ulcerans* showed median cytotoxicity of 20%, suggesting that cytotoxicity observed in *M. ulcerans*-infected mice was due to the presence of a cytotoxic molecule with activity similar to, or targeting the same pathway as, mycolactone.

The mean concentration, as estimated in the cytotoxicity assay, of mycolactone in the right footpad (3 per group) increased from 286±105 ng/ml at day 3 to 948±215 ng/ml at day 7, plateaued between day 13 and 35 and progressively increased to 3603±478 ng/ml at day 62 (Figure 5). In the left footpad of mice infected on the right, the concentration also increased up to day 62 with a peak of 1524±607 ng/ml at day 27. In the right and left footpads of antibiotic treated mice, there was a significant decline in estimated mycolactone concentration compared to untreated right footpads (p<0.05 at day 62). Likewise, there was a significant difference (p<0.02) in cytotoxicity between the right and the contralateral footpads obtained from untreated mice.

#### Mass spectroscopic analysis

**Figure 6:**
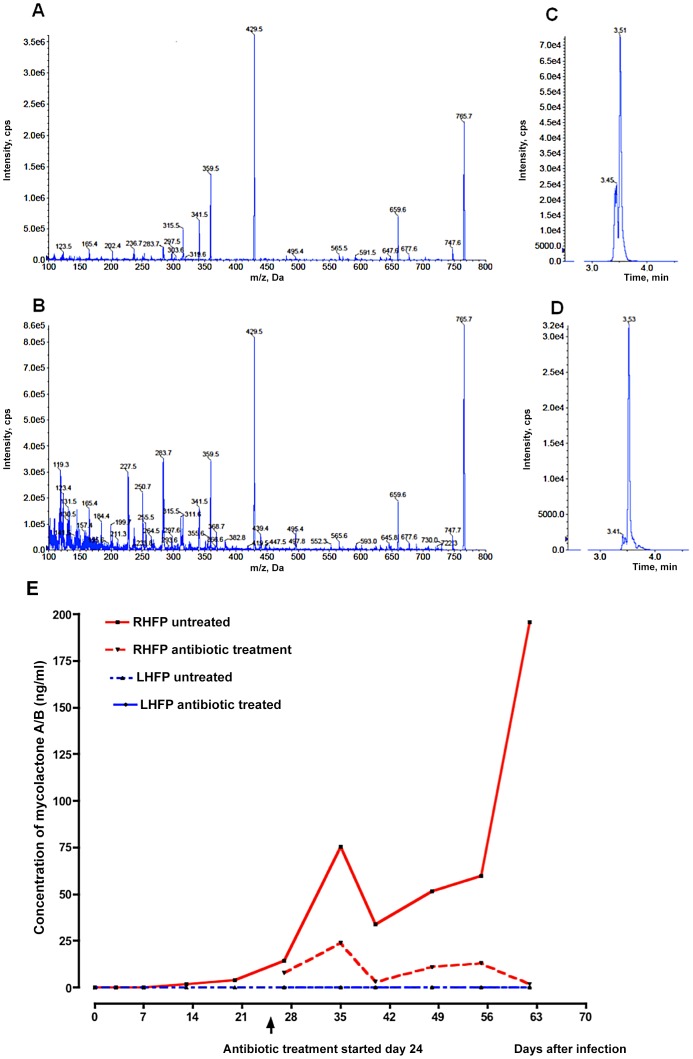
Mycolactone detection by mass spectrometry in mouse footpads. A. EPI Mass spectra indicated a propensity of authentic mycolactone A/B to form a sodium adduct (m/z 765.7, [M+Na+]). Fragment ions at m/z 429.6 and 359.5 correspond to the core lactone and polyketide side chains, respectively. B. Mycolactone A/B detected in footpad extracts displayed similar mass spectra and also contained a number of other ions (e.g. m/z 659.6 and 747.7) that were present in authentic reference material. C and D. Extracted ion chromatograms (XIC) for m/z 429.6, which was selected for quantification in LC-MRM analyses of tissue extracts. Standard mycolactone is shown in C and a footpad sample in D. The sensitivity of the assay (<10 pg) was such that it was possible to measure mycolactone in the tissue samples. E. Mycolactone (ML) was detectable as early as day 13 after infection and before the onset of visible swelling in mouse footpads. No ML was detectable in the contralateral footpad of either the RIF-STR-treated or the untreated control mice. Data based on pooled lipid extracts from 3 mice per group per time point.

In order to establish whether cytotoxicity in infected mouse footpads was due to whole mycolactone molecules, liquid chromatography was coupled to mass spectroscopic detection of mycolactone A/B. Using an enhanced product ion (EPI) method, mycolactone A/B was detected as an ion with m/z of 765.7 which fragmented to give peaks at 429.5 (core lactone ring), 359.5 (polyketide side chain) and other minor peaks at 747.7, 659.6 and 565.6 (Figure 6 A–D). Using this approach, intact mycolactone A/B was identified in the infected right footpad pools at day 13 onwards but not in the uninfected left footpads.

An approximate quantification of mycolactone A/B was achieved using multiple reaction monitoring (MRM). (Figure 6E). In the infected right footpads, mycolactone A/B concentration increased from 1.6 ng/ml on day 13 to a peak of 195.6 ng/ml on day 62. In antibiotic treated mice mycolactone concentration in the right footpads decreased from a peak of 23.7 ng/ml on day 35 to 1.6 ng/ml on day 62. No mycolactone was detected in left footpads.

### Conclusions

Our results show that RS treatment of experimental *M. ulcerans* disease results not only in the reduction or elimination of viable bacteria but also in clinical and histopathological improvement of lesions, supporting results obtained in the analysis of human specimens [22], [23] and in RS-treated mice [24], [25], [26]. The inflammatory response is also down-regulated with reduced production of cytokines such as TNF-α during treatment. By mass spectrometric analysis, mycolactone was detectable before the observation of swelling and increased markedly and continuously after swelling onset whereas *M. ulcerans* CFU counts peaked and remained on a plateau. RS treatment resulted in a parallel loss of cultivable *M. ulcerans* in the footpad lesions, a decline in footpad swelling and an abrupt decline in the production of mycolactone within days of treatment initiation.

## Discussion

The initial clinical response to infection with *M. ulcerans* is typically not strikingly different from the course of infection with *M. marinum* [27], [28], [29], [30]; a nodule develops in both cases but, with *M. ulcerans*, there is an absence of tenderness and erythema. The absence of pain and an inflammatory response is attributed to the production of mycolactone inhibiting the immune response [31], damaging nerves [32], [33] and/or destroying infiltrating inflammatory cells [34], [35]. At which point in the course of infection sufficient mycolactone is present to mediate these effects cannot be readily ascertained in humans but can be approached in the mouse model. The mouse model is also suitable to monitor the impact on mycolactone production of antibiotic treatment, which is thought to block mycolactone production and to reverse local immunosuppression leading to the restoration of an active inflammatory process [36].

In the experiment reported here, mycolactone was detectable 2 to 3 weeks after inoculating approximately 4.6 log_10_ CFU of *M. ulcerans* into mouse footpads and before detectable swelling of the footpads. The numbers of bacilli at the beginning of a human infection are unknown but presumably considerably less. A sensitive assay that would be appropriate for use in endemic settings could conceivably detect infection before the nodular phase or confirm the diagnosis of *M. ulcerans* infection in nodular or plaque lesions before the onset of ulceration. Treatment with the current gold standard of rifampin and streptomycin appears to result in inhibition of mycolactone production. Whether the inhibition blocks the pathway of enzymes encoded by the *M. ulcerans* giant plasmid or has an impact on the plasmid itself has not been assessed here but it could be investigated using samples where there are still cultivable bacilli but negligible mycolactone production. Mechanistically, streptomycin and rifampicin inhibit protein synthesis by different mechanisms but they both result in a decrease in bacterial viability and, directly or indirectly, in a decrease of mycolactone production.

By sampling mouse footpads on a weekly basis we have been able to confirm the early phagocytic, intracellular phase of *M. ulcerans* infection followed by a later extracellular phase [26]. One week after infection (Fig. 3A2), there was moderate infiltration of host cells and bacilli were predominantly intracellular, an observation made by Ruf et al. [25]. The extracellular phase of bacillary growth appeared to coincide with the onset of footpad swelling, beginning about 3 weeks after infection in this experiment. Over the next two weeks, AFBs were increasingly found outside of cells. The shift to an extracellular growth was blocked by treatment with rifampin and streptomycin, consistent with the notion that mycolactone production and phagocyte destruction are inhibited by antibiotic treatment [22], [23], [24]. In the absence of treatment, the location of bacterial clusters also changed from relatively deep in the dermis to sub-epidermal and epidermal zones to the skin surface with the onset of footpad ulceration. In contrast, in mice treated with the antibiotics, *M. ulcerans* was found in a few clusters associated with phagocytic cells within the dermis. The appearance of the bacteria became beaded with less intense acid-fast staining [22], [23], [24], [25], comparable to the changes observed in the morphological index of leprosy bacilli after the onset of treatment. At 3 months after the completion of 8 weeks of daily treatment, pale-staining bacilli were still detectable and associated with host cells in the dermis but remained uncultivable, suggesting that residual antigenic components may have helped maintain an inflammatory infiltrate. Overall, from the literature [25], [34], [37], we find that early after infection, there is a dynamic picture of infiltrating host cells and bacillary localization within phagocytic cells. When the same time points were examined in mouse footpads on day 7 (this study and [25]) and day 13 (this study and [34]), the histological impressions were strikingly similar.

In this experiment, we observed increases in *M. ulcerans* CFU counts starting at day 3 (3.29±0.33 log_10_) and continuing to day 27 (6.02±0.13 log_10_) after infection. The CFU counts remained at approximately 6 log_10_ for the duration of the experiment through day 55, after which time the footpads started to ulcerate and could not be readily decontaminated for further analysis, given the prolonged culture time for this organism (8–12 weeks). Swelling was not apparent until day 20 in some mice, but by days 24 and 27, it averaged grade 1. Swelling continued to increase during this time, while CFU counts were stable, and peaked only at day 55 at an average of swelling grade 3.75. The fact that mycolactone concentration continued to rise after the plateau in cultivable organisms suggests that there was equilibrium between living and dying *M. ulcerans* cells with mycolactone being released by both populations. Alternatively, or in addition, mycolactone may be induced by a quorum sensing mechanism as bacterial numbers reach the plateau stage. Concurrently, or slightly before the manifestation of swelling, mycolactone could be detected by mass spectrometric analysis at day 13 (1.6 ng/ml of homogenized footpad extract) and 4.18 ng/ml on day 20. In untreated mice the concentration of mycolactone in infected footpads continued to rise throughout the experiment. In mice treated with antibiotics from day 24, mycolactone concentration tailed off after day 35 but it did not become undetectable by MS until day 63. This suggests either that viable organisms persisted for more than 5 weeks after antibiotics were started or that mycolactone could still be detected some time after *M. ulcerans* had been killed. The latter is supported by the observations that no viable *M. ulcerans* were detected in mouse footpads after antibiotic treatment, similar to that used here, followed by a course of corticosteroid therapy [24], [38] but we did not use the very recently described 16S rRNA assay to determine if viable organisms were still present [39].

Using the cytotoxicity assay, biological activity associated with mycolactone was detectable as early as day 3 and persisted even at day 63 despite antibiotic treatment. Although it is possible that this assay is more sensitive than MS, experiments in vitro using synthetic mycolactone as a standard show that MS can detect mycolactone at a concentration of less than 10 pg/ml so it is more likely that some cytotoxicity was caused by other lipid molecules generated during the course of *M. ulcerans* infection. Nevertheless this evidence that mycolactone may have persisted in mouse tissue well after antibiotics had started to reduce the burden of infection when footpad swelling was diminishing is important in the context of human infection in which slow healing has been observed in some cases without an obvious reason.

Mycolactone has profound effects on the production of cytokines and chemokines in vitro even at sub-cytotoxic concentration and this may well influence the rate of wound healing. In the present experiments for example, TNF-α production by splenocytes increased in response to mycobacterial antigens during *M. ulcerans* infection but this reverted to normal during antibiotic treatment. A similar but delayed and relatively attenuated response was observed for other cytokines such as IFN-γ, IL-5, and IL-17. ConA, a potent mitogen, assessed the capacity of splenocytes to produce cytokines upon stimulation, while the Mtb CFP elicited responses to mycobacterial antigens but is not specific for *M. ulcerans*. The initial rise in the secretion of cytokines coincides with the infiltration of host inflammatory cells and the intracellular stage of *M. ulcerans* when mycolactone concentrations were low. Subsequently mycolactone concentrations start to build up at the site of infection, *M. ulcerans* assumes an extracellular localization and prevents antigen-presenting cells from processing mycobacterial antigens for a systemic immune response. The fact that *M. ulcerans* assumes an extracellular localization and that mycolactone concentrations begin to increase locally could prevent antigen presenting cells from processing *M. ulcerans* for systemic immune responses to develop [40]. Again Hong et al. [41] have shown that mycolactone A/B could be detected in PBMC's of mice infected with *M. ulcerans* and indeed recently ML has been detected in sera of some human's infected with *M. ulcerans* [42]. The later recovery of systemic cytokine secretion in the light of increasing concentrations of mycolactone locally in the footpads is difficult to explain. In the proximal phases of *M. ulcerans* infection, this initial exposure may be inhibited by increasing bacillary load and the production of mycolactone. Subsequently, the splenocytes could encounter and process mycobacterial antigens released into the circulation or conveyed from the site of infection in the later stages of *M. ulcerans* infection from degradation of defunct *M. ulcerans* in mice treated with bactericidal antibiotics (Figure 4, both columns).

Some patients are able to control *M. ulcerans* infections at the early nodular stage without antibiotics and these data may provide some insight into why this might be the case. It would seem that those with robust immune responses, a lower burden of *M. ulcerans* and thus mycolactone secretion initially may be able to contain the *M. ulcerans* infection. Further studies are, however, required to elucidate the correlations between local and systemic mycolactone kinetics in tandem with immune responses in these two compartments in this model.

The finding that cytotoxicity was increased in contra-lateral footpads well away from the site of infection remains difficult to explain. Mycolactone molecules were not detected by MS but we cannot rule out the possibility that cytotoxic breakdown products of mycolactone, or other molecules, circulated to these footpads. Other studies have shown that mycolactone itself was detectable in peripheral blood white cells during mouse infection with *M. ulcerans* [41].

Further studies may focus on the determination of cytokines in the milieu of the footpad lesion in mice before and after treatment with the current regimen or a new all oral regimen, such as one replacing streptomycin with clarithromycin, as well as the impact of vaccination on mycolactone production and local immunity after footpad challenge. Refinements in calculating the amount of mycolactone obtained after extraction may be made by spiking different concentrations of synthetic mycolactone into uninfected footpads and determining the linearity of the yield.
